# Rare inventory of trematode diversity in a protected natural reserve

**DOI:** 10.1038/s41598-021-01457-2

**Published:** 2021-11-11

**Authors:** Jessica Schwelm, Christian Selbach, Jenia Kremers, Bernd Sures

**Affiliations:** 1 Aquatic Ecology and Centre for Water and Environmental Research, University of Duisburg-Essen, Universitätsstraße 5, 45141 Essen, Germany; 2 Department of Biology, Aquatic Biology, Aarhus University, 8000 Aarhus C, Denmark; 3 Department of Zoology, University of Johannesburg, Johannesburg, South Africa

**Keywords:** Biodiversity, Freshwater ecology

## Abstract

In the face of ongoing habitat degradation and the biodiversity crisis, natural reserves are important refuges for wildlife. Since most free-living organisms serve as hosts to parasites, the diverse communities in protected areas can be expected to provide suitable habitats for a species-rich parasite fauna. However, to date, assessments of parasite diversity in protected nature reserves are rare. To expand our knowledge of parasite communities in natural habitats, we examined 1994 molluscs belonging to 15 species for trematode infections in a central European natural reserve. The parasitological examination revealed an overall prevalence of 17.3% and a total species richness of 40 trematode species. However, the parasite diversity and prevalence did not differ markedly from trematode communities in non-protected environments, which might be partly explained by a dilution effect caused by a high number of non-host organisms in our study system. The proportion of complex and long life cycles of parasites in the present study is high, indicating complex biotic interactions. We conclude that life cycle complexity, in addition to parasite diversity and trematode species richness, can provide valuable information on ecosystem health and should therefore be considered in future studies.

## Introduction

Anthropogenic activity has a direct impact on most of the Earth’s ecosystems. Globally, human activities have altered a large part of the terrestrial area^1^, the oceans^2^ and freshwater habitats^3^. The latter are among the most affected and threatened ecosystems suffering from multiple stressors^3^. The ongoing freshwater biodiversity crisis is gaining increasing awareness as a major environmental problem, as exemplified by implementation of the European Water Framework Directive (WFD)^4^. Mainly due to habitat degradation, an increasingly large number of species is threatened by extinction^5,6^. More frequent and more extreme weather events, such as droughts, storms or floods in the context of the global climate change, deterioration of freshwater quality, and the rapidly growing global population will further intensify this problem^7–10^. Natural reserves and protected areas are therefore becoming increasingly important as reservoirs and retreat areas for aquatic organisms, including rare and endangered species which are considered worthy of protection by society and politics^11,12^. These groups of organisms are well documented in many systems (e.g.^13,14^) and are used, for instance, as indicator species for the environmental conditions of ecosystems^15,16^.

Free-living organisms may serve as hosts for a wide range of parasitic species. Since most free-living animals are assumed to host at least one parasite species, a correspondingly high diversity of parasites must be expected in natural reserves or protected areas. Parasitism is considered as one of the most successful and widespread life forms, and estimates of parasite contributions to global species diversity range from 30 to 50%^17–19^. Although parasites are often overlooked (or ignored) in nature conservation approaches and discussions, they play crucial roles in every ecosystem, influencing and shaping it in various ways. For instance, parasites have been shown to act as ecosystem engineers^20^, shaping and regulating host population dynamics^21–23^ as well as predator prey interactions^24,25^ by influencing host growth, mortality, fitness and behaviour^26–28^. Parasites therefore have to be considered as important structuring forces in food-webs^29,30^. Furthermore, they account for a large part of the biomass of ecosystems and contribute significantly to the energy flow within those systems^31–34^. Due to their complex life cycles, parasites may also function as bioindicators to determine environmental conditions and changes^35–37^.

With about 25,000 described species and a cosmopolitan distribution, digenean trematodes represent one of the most diverse and widespread groups of metazoan parasites on the planet^38^. Trematodes have complex life cycles, with molluscs, mostly gastropods, playing a key role as first intermediate hosts. Besides vertebrates as obligate final hosts, a broad spectrum of invertebrates and vertebrates serve as second intermediate hosts. Due to the trophic transmission of many trematode species, they are particularly interwoven into the food-web and energy flow within ecosystems. Moreover, knowledge of the occurrence of specific trematodes in a habitat can provide valuable information on the presence of the hosts required by the parasite (see e.g.^39^), meaning that trophically transmitted parasites in particular can act as cross-taxon surrogates for the presence of their hosts^40^.

Encouragingly, topics such as parasite extinction and conservation have recently gained more attention (e.g.^41–44^). Even a global plan for their protection and incorporation in monitoring programs has been proposed lately^42^. Due to their lifestyle, parasites are especially vulnerable and threatened with extinction, either when they are directly affected^42^ by factors such as climate change or invasive species^45^, or indirectly via the extinction of their hosts^46,47^. Although the importance of parasites in ecosystems has been increasingly recognised (e.g.^20–22,48,49^), we still face the problem that knowledge about the distribution of many parasite taxa is fairly incomplete^50^. In contrast to well-studied “man made” and anthropogenically influenced freshwater systems (e.g.^51–54^), our knowledge of these parasites in natural systems is often still limited. This is especially true for protected areas, since such habitats are increasingly rare and usually not easily accessible. Due to restrictions and protection measures, assessments of parasite biodiversity in such areas encounter various obstacles and are inevitably associated with high administrative burdens. This shortcoming remains a fundamental obstacle, as the knowledge on trematode diversity in protected areas might represent the best approximation of a natural status of the parasites in these regions, which can be used as a basis to assess changes in trematode community composition in heavily modified ecosystems. In the absence of this basic information, it will also be difficult to predict whether and how parasites will react to the ongoing anthropogenic-driven habitat alterations and how these reactions will affect ecosystem processes.

We hypothesize that freshwater natural reserves serve as a home to diverse, species-rich and well-connected communities of digenean trematodes, reflecting the system's free-living species richness. We predict parasites in natural reserves to (i) show species-rich communities that (ii) predominantly contain parasite species with complex life cycles (i.e. more hosts involved in the life cycle) and (iii) a high proportion of trematodes parasitizing rare host taxa. The aim of the present study is to assess the diversity of trematodes in a protected European freshwater natural reserve and to relate these data to other well-studied freshwater systems.

## Results

### Occurrence of snails and trematodes

**Figure 1:**
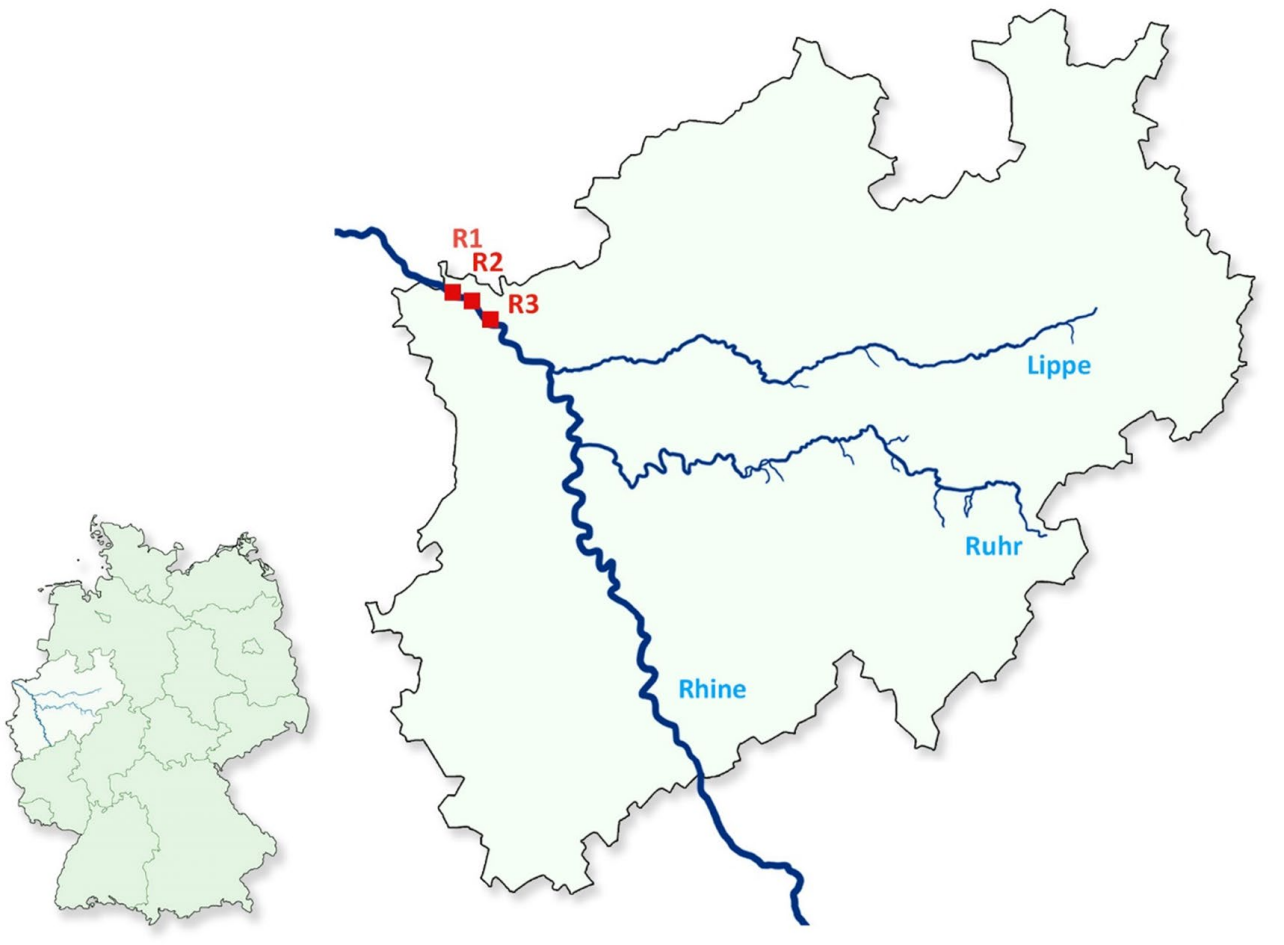
Map of Germany (left) and the federal state of North Rhine-Westphalia (right) indicating the sampling sites at the natural reserve Bienener Altrhein. Sampling sites are marked with red squares.

A total of 1994 snails was collected at three different sites during monthly samplings from May to October 2017 and from May to September in 2019 in the natural reserve “Bienener Altrhein” in North Rhine-Westphalia, Germany (Fig. 1). Fifteen snail species belonging to seven families were sampled (Bithynidae, Hydrobiidae, Lymnaeida, Physidae, Planorbidae, Valvatidae, Viviparidae). Nine of these species harboured larval trematodes. The lymnaeid *Ampullaceana balthica* (former name: *Radix balthica*), which was collected in 2017 and 2019, was the most frequently collected snail species (n = 574), followed by the planorbids *Planorbis planorbis* (n = 309) and *Planorbarius corneus* (n = 228) (Table 1).

**Table 1:** Overview of collected mollusc species, number and prevalence of infection, and the number of harboured trematode species.

Collected mollusc species	Total no. of snails	Total no. of infections	Overall prevalence of infection (%)	No. of harboured trematode species
**Bithynidae**				
*Bithynia tentaculata*	75	14	18.7	11
**Hydrobiidae**				
*Potamopyrgus antipodarum*	49	–	–	–
**Lymnaeidae**				
*Lymnaea stagnalis*	160	32	20	9
*Ampullaceana balthica*	574	183	31.9	13
*Stagnicola palustris*	22	13	59.1	1
**Physidae**				
*Aplexa hypnorum*	87	–	–	–
*Physa acuta*	143	1	0.7	1
**Planorbidae**				
*Anisus vortex*	137	4	2.9	1
*Bathyomphalus contortus*	16	–	–	–
*Ferrissia fragilis*	15	–	–	–
*Gyraulus albus*	16	–	–	–
*Planorbarius corneus*	228	65	28.5	10
*Planorbis planorbis*	309	22	7.1	4
**Valvatidae**				
*Valvata piscinalis*	141	11	7.8	5
**Viviparidae**				
*Viviparus viviparus*	22	–	–	–
Total	1994	345	17.3	40

**Figure 2:**
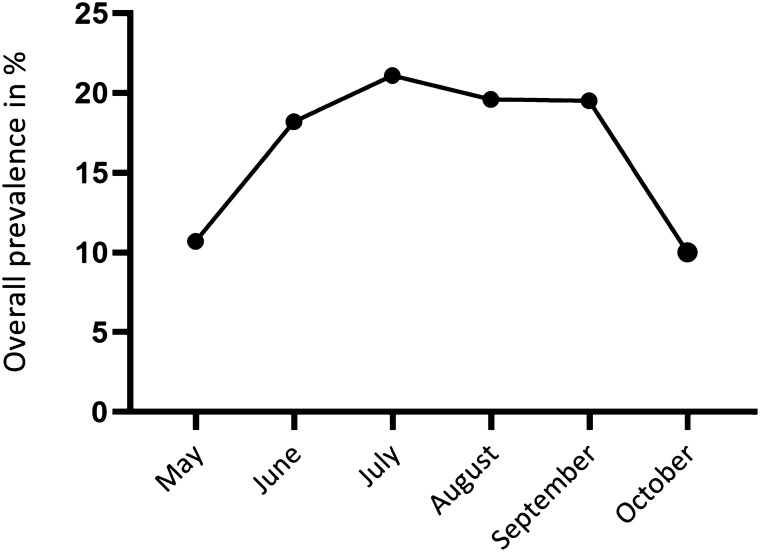
Seasonal overall trematode prevalence pooled from all collected snails in 2017 and 2019.

**Figure 3:**
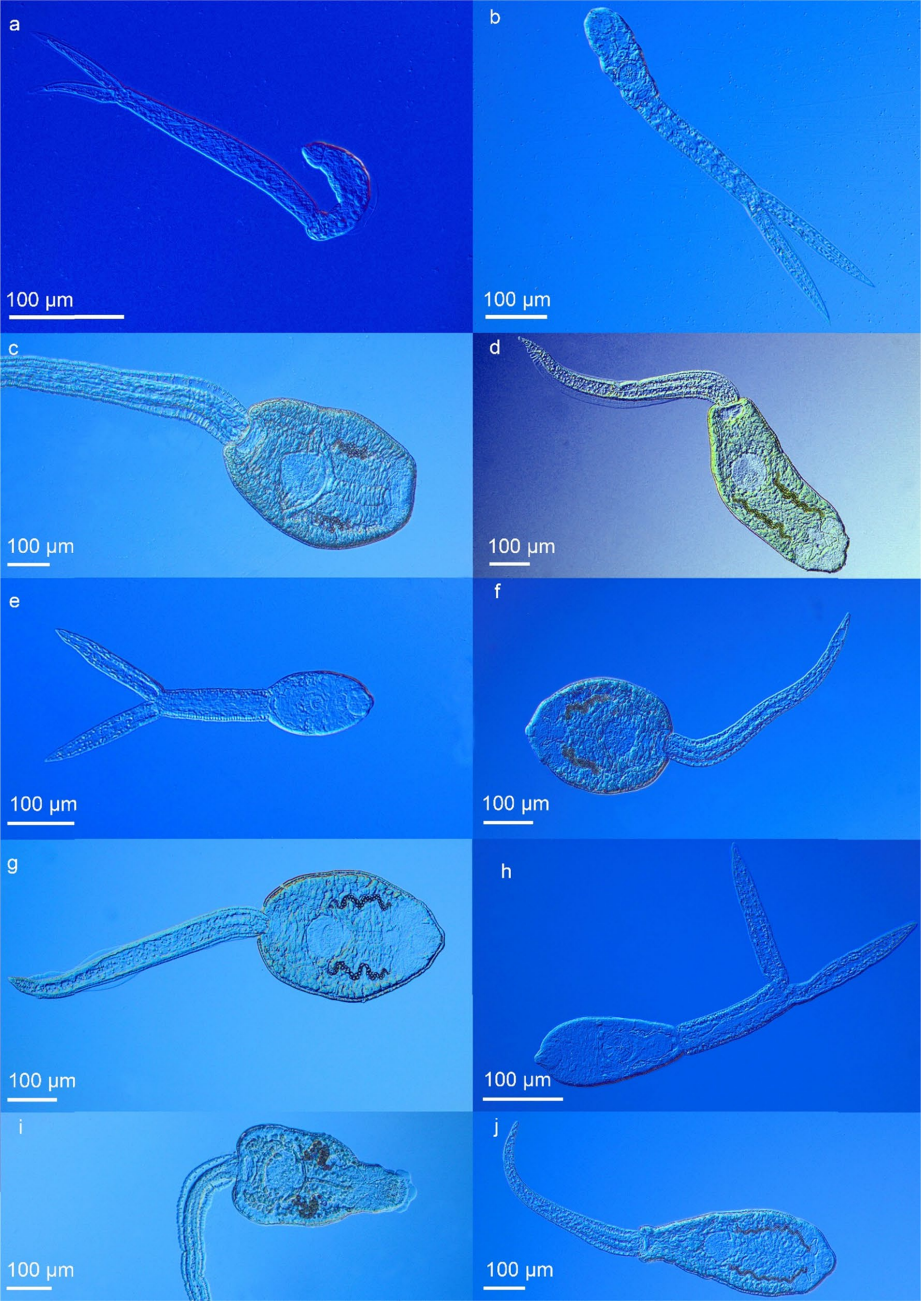
Microphotographs of live cercariae of the 10 most prevalent species. (**a**) *Sanguinicola inermis* (found 7 times). (**b**) *Tylodelphis excavata* (8). (**c**) *Echinoparyphium aconiatum* (10). (**d**) *Moliniella anceps* (14). (**e**) *Australapatemon burti* (16). (**f**) *Hypoderaeum conoideum* (19). (**g**) *Echinostoma revolutum* (38). (**h**) *Cotylurus* sp. (45). (**i**) *Petasiger radiatus* (60). (**j**) *Echinoparyphium recurvatum* (64).

Parasitological examination of molluscs revealed an overall prevalence of 17.3% and a total species richness of 40 digenean trematode species. Distinct seasonal variations occurred among the sampling periods (χ^2^ test, Df = 5, n = 1,994, χ^2^ = 21.7, p < 0.001), with overall prevalence peaking in the summer months compared to spring and early autumn (Fig. 2). In total, 15 trematode families were identified. The three most species-rich parasite families in our study represent the Echinostomatidae (10 species), Diplostomidae (5) and Strigeidea (4). The most abundant trematode species detected were *Echinoparyphium recurvatum* (found 64 times), *Petasiger radiatus* (60), *Cotylurus* sp. (45), *Echinostoma revolutum* (38) and *Hypoderaeum conoideum* (19) (Fig. 3).

With 13 trematode species, *A. balthica* showed the highest species richness among the snail hosts studied, followed by *Bithynia tentaculata, P. corneus* and *Lymnaea stagnalis* with 11, 10 and 9 species, respectively. The remaining snail hosts harboured only few species (Table 1). The relative species richness is on average four trematode species per snail species (Table 2).

**Table 2:** Overview of snail host species, trematode species richness and prevalence from comparable well-studied fresh water systems in Europe and North America.

Sampling sites	Country	No. of snails species	No. of snails species showing infections	No. of snail sampled	No. of trematode species	Relative species richness	Overall prevalence (%)	Reference
Central European natural reserve	Germany	15	9	1994	40	4.4	17.3	Present study
Northern European lake	Norway	3	2	1007	15	7.5	21.5	^57^
Northern American lakes	Canada	5	5	13,179	39	7.8	13.5	^56^
Central European fish ponds	Czech Republic	11	11	2584	27	2.5	15.2	^51^
Central European lakes	Poland	6	6	10,527	25	4.2	35.1	^52^
Central European reservoirs	Germany	6	6	5347	36	6.0	19.6	^54^

### Trematode life cycle complexity

**Figure 4:**
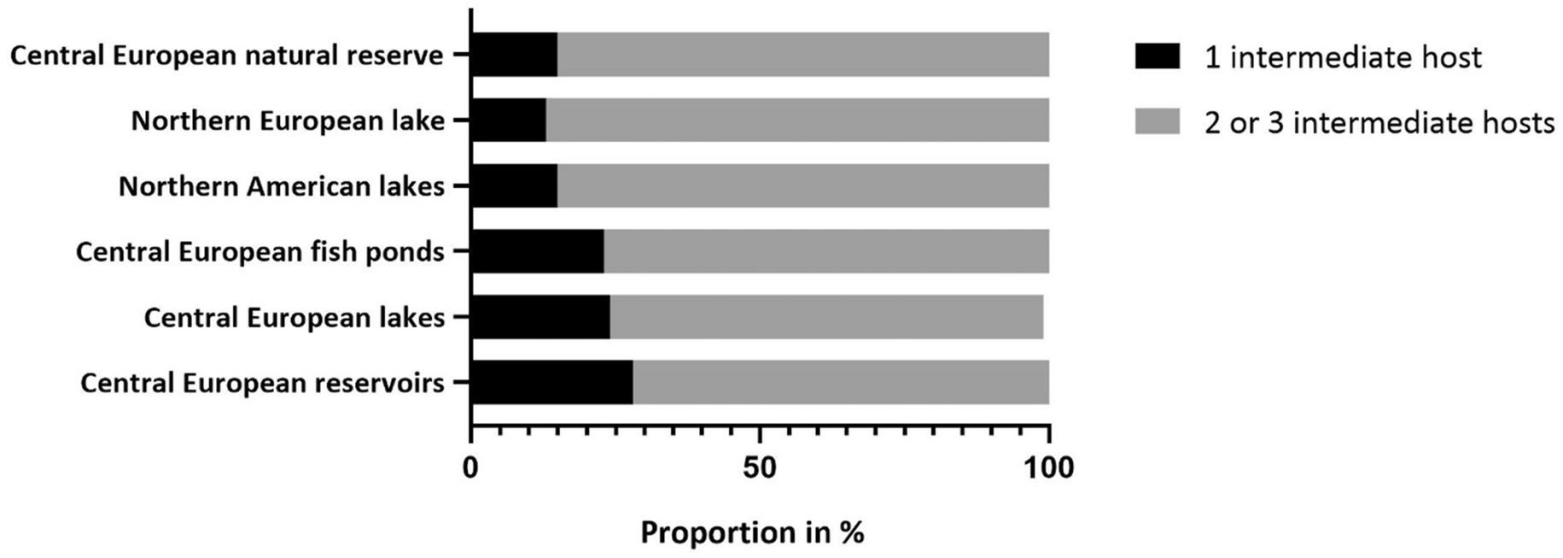
Proportion of trematode species with one intermediate host and two or more intermediate hosts for the studied Central European natural reserve in comparison to five freshwater systems with varying degree of anthropogenic impact.

Based on a literature survey, the life cycles of the trematode species identified in this study were reconstructed. Our investigation revealed that the proportional occurrence of detected digenean trematodes with a shorter life cycle (i.e. one intermediate host) accounts for 15%, compared to 85% with longer life cycles (i.e. two or three intermediate hosts) (Fig. 4). Species of the genera *Asymphylodora*, *Notocotylus*, *Sanguinicola* and *Trichobilharzia* are the main representatives that require only one intermediate host. Species belonging to the genera *Asymphylodora* and *Sanguinicola* utilise fish as final hosts, whereas species of the genera *Notocotylus* and *Trichobilharzia* parasitize waterfowl (Table 3). No significant association between sampling location and life cycle length were detected (Fisher’s exact test, *p* = 0.648).

**Table 3:** Summary of the recorded trematode species in all collected mollusc species and their second intermediate and final host groups.

Trematode family and species	First intermediate host*	Second intermediate host	Final hosts (literature data)	Final hosts (classification for analysis)	No. of required hosts	References
**Cyathocotylidae**						
Cyathocotylidae gen sp.	BT	Fishes, amphibians, aquatic invertebrates	Reptiles, birds, mammals	Reptiles, birds, mammals	3	^85^
*Cyathocotyle* sp. 1	BT	Fishes, amphibians, aquatic invertebrates	Reptiles, birds	Reptiles, birds, mammals	3	^85^
*Cyathocotyle* sp. 2	BT	Fishes, amphibians, aquatic invertebrates	Reptiles, birds	Reptiles, birds, mammals	3	^85^
**Diplostomidae**						
*Alaria* sp.	PP	Frogs, amphibians	Carnivores	Mammals	3 or 4^a^	^86^
*Diplostomum pseudospathaceum*	LS	Fishes	Fish-eating birds	Fish-eating birds	3	^61,87^
*Diplostomum* sp.	AB	Fishes	Fish-eating birds	Fish-eating birds	3	^88^
*Hysteromorpha triloba*	AV	Fishes	Fish-eating birds: cormorants, herons, grebes	Fish-eating birds	3	^61,88^
*Tylodelphis excavata*	PC	Amphibians	Birds: storks, herons, birds of prey	Birds	3	^61,87^
**Echinochasmidae**						
*Echinochasmus coaxatus*	BT	Fishes	Birds, fish-eating birds: grebes	Fish-eating birds	3	^89^
*Echinochasmus bursicola*	BT	Fishes	Fish-eating birds: herons	Fish-eating birds	3	^89,90^
*Echinochasmu*s sp. 1	BT	Unknown	Fish-eating birds	Fish-eating birds	3	^89^
**Echinostomatidae**						
*Echinoparyphium aconiatum*	LS, AB	Molluscs, tadpoles	Anatidae	Waterfowl	3	^87^
*Echinoparyphium recurvatum*	LS, AB, PP, VP	Molluscs, tadpoles	Anatidae	Waterfowl	3	^87^
*Echinostoma revolutum*	LS, AB	Molluscs	Anatidae	Waterfowl	3	^87^
*Echinostoma* sp.	PC	Molluscs, bivalves, planarians, tadpoles	Waterfowl	Waterfowl	3	^87^
*Hypoderaeum conoideum*	AB	Molluscs	Waterfowl	Waterfowl	3	^87^
*Moliniella anceps*	AB, SP	Molluscs	Rallidae	Birds	3	^87^
*Moliniella* sp.	VP	Molluscs	Rallidae	Birds	3	^87^
*Neoacanthoparyphium echinatoides*	VP	Molluscs	Birds, Anatidae	Waterfowl	3	^91^
*Neopetasiger* sp. 3	PP	Tadpoles, Fishes: Cyprinids	Grebes	Fish-eating birds	3	^92^
*Petasiger radiatus*	LS, AB, PA, PC, PP	Fishes	Cormorants	Fish-eating birds	3	^61,87,92^
**Lissorchiidae**						
*Asymphylodora* sp.	VP	None (direct life cycle)	Fishes	Fishes	2	^93^
**Notocotylidae**						
*Notocotylus attenuatus*	LS; PC	None (cercariae encyst on vegetation)	Anatidae	Waterfowl	2	^87^
*Notocotylus ephemera*	PC	None (cercariae encyst on vegetation)	Anatidae	Waterfowl	2	^87^
**Omphalometridae**						
*Rubenstrema* sp.	PC	Insect larvae	Mammals	Mammals	3	^87^
**Plagiorchiidae**						
*Plagiorchis elegans*	LS; PC	Molluscs insect larvae, freshwater crustaceans	Various birds, mammals	Reptiles, birds, mammals	3	^87,94^
*Plagiorchis neomidis*	AB	Molluscs, insect larvae, freshwater crustaceans	Various birds, mammals: Soricidae	Reptiles, birds, mammals	3	^94^
**Pleurogenidae**						
*Pleurogenidae* gen sp. 2	BT	Unknown	Waterfowl: Rallidae	Birds		^95^
**Prosthogonomidae**						
*Prosthogonimus ovatus*	BT	Insect larvae	Birds	Birds	3	^96^
**Psilostomidae**						
*Sphaeriodiotrema* sp.	BT	Molluscs	Waterfowl	Waterfowl	3	^97^
Psilostomidae gen sp. 1	BT	Unknown	Birds, mammals	Reptiles, birds, mammals	3	^98^
Psilostomidae gen sp. 2	BT	Unknown	Birds, mammals	Reptiles, birds, mammals	3	^98^
**Sanguinicolidae**						
*Sanguinicola inermis*	AB	None (direct life cycle)	Fishes: Cyprinids	Fishes	2	^61,87^
*Sanguinicola* sp.	VP	None (direct life cycle)	Fishes	Fishes	2	^61,87^
**Schistosomatidae**						
*Trichobilharzia szidati*	LS	None (direct life cycle)	Anatid birds	Waterfowl	2	^87^
**Strigeidae**						
*Australapatemon burti*	AB; VP	Leeches	Anatid birds	Waterfowl	3	^87^
*Australapatemon* sp.	AB	Unknown	Anatid birds	Waterfowl	3	^99^
*Cotylurus cornutus*	LS	Leeches, Molluscs	Anatid birds	Waterfowl	3	^87^
*Cotylurus* sp.	PC	Leeches, Molluscs	Anatid birds	Waterfowl	3	^87,99^
**Telorchiidae**						
*Opisthioglyphe ranae*	AB	Amphibians, tadpoles	Amphibians: Anura	Amphibians	3	^87^

*BT: *Bithynia tentaculata*; LS: *Lymnaea stagnalis*; AB: *Ampullaceana balthica*; SP: *Stagnicola palustris*; PA: *Physa acuta*; AV: *Anisus vortex*; PC: *Planorbarius corneus*; PP: *Planorbis planorbis*; VP: *Valvata piscinalis*.

^a^*Alaria* spp. develop in a three-host life cycle with an interjectional mesocercarial stage between the cercarial and the metacercarial stage. This life cycle can be extended by paratenic hosts.

### Host taxa

**Figure 5:**
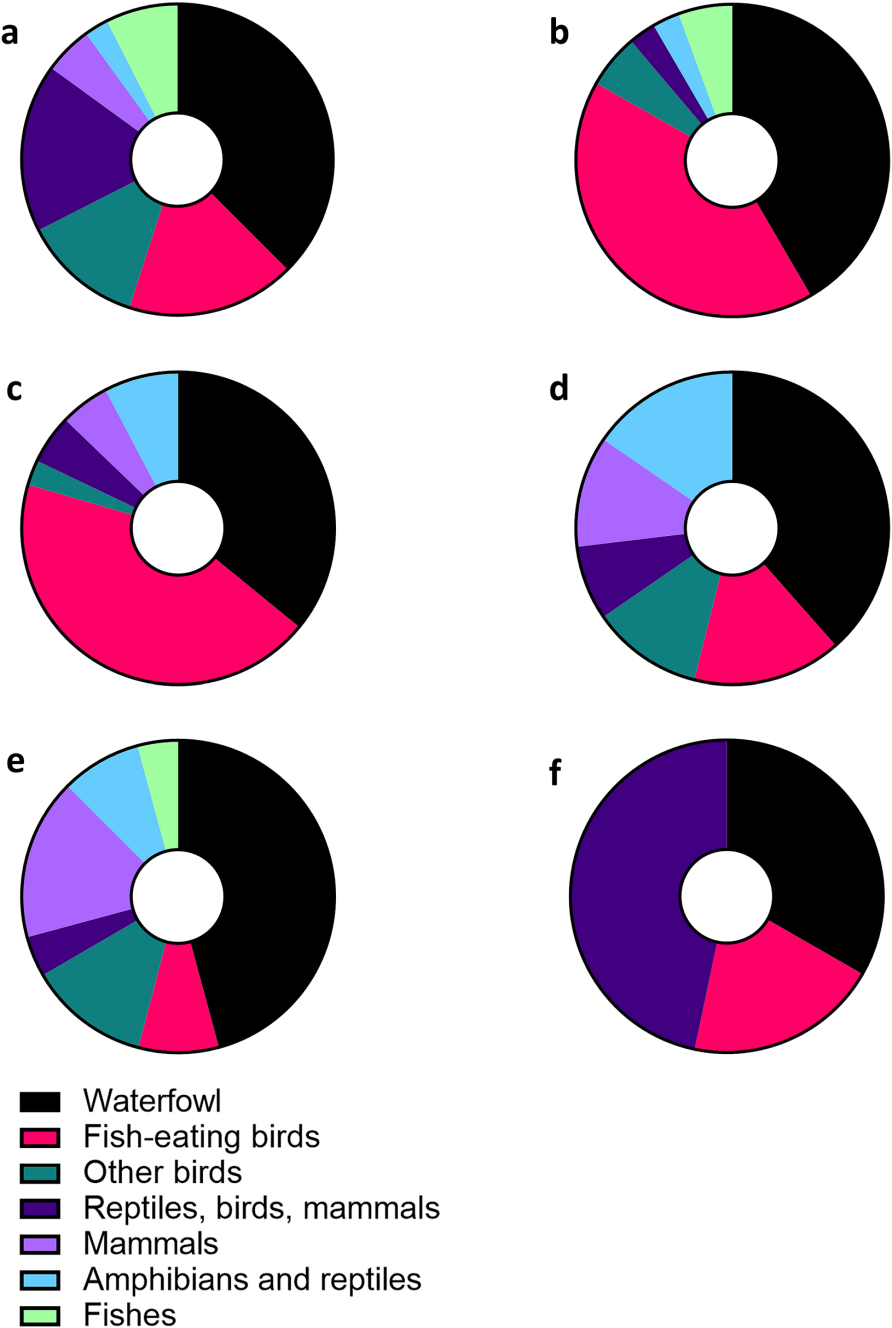
Proportion of final hosts. (**a**) Central European natural reserve. (**b**) Central European reservoirs. (**c**) Northern American lakes. (**d**) Central European fish ponds. (**e**) Central European lakes. (**f**) Northern European lake.

An extensive literature search was conducted to compile an overview of all recorded trematode species in their second intermediate and final host groups (Table 3). We reviewed final host specificity to detect trematode species that use rare and/or protected host species. Additionally, we looked at host specificity regarding the first intermediate host, for which trematodes usually exhibit high specificity^55^. With a few exceptions, individual trematode species were mainly found in one snail host species or in different species of the same family. The exceptions include, for instance, *P. radiatus or E. recurvatum*, which were detected in five and four snail species from three families, respectively. A large proportion of digeneans parasitize waterfowl (37.5%), in most cases anatid birds. Both, fish-eating birds and a very unspecific group consisting of mammals, birds and amphibians each account for almost 18% of hosts (Fig. 5).

## Discussion

Our present knowledge of parasite diversity is still very limited and reveals major gaps in our understanding of the distribution of parasites in different ecosystems, especially in natural freshwaters. Due to this incomplete knowledge of parasite diversity, predictions about how anthropogenic environmental changes will affect parasite communities in the future remain difficult. The present study therefore presents a valuable and rare inventory of trematode diversity in a protected and natural aquatic system and is, to the best of our knowledge, the first evaluation of trematode communities in a natural freshwater reserve. While we can confirm our hypothesis that the studied freshwater nature reserve supports diverse and species-rich trematode communities, no significant differences in parasite life cycle complexity could be shown in comparison to other habitats, and the patchy data basis on host specificity of digenean trematodes hinders accurate conclusions on the role of rare host taxa in our study system.

With 40 trematode taxa in 1994 snails, the trematode species richness in our system ranks at the top compared to similar faunistic surveys from freshwater systems^51,52,54,56,57^. However, due to the rather high number of snail host species analyzed in the present study (15 species), of which some hosts harboured only few or no parasites, our study area shows a rather medium to low average species richness per host species with four trematode species per snail species. Moreover, with a prevalence of 17.3%, the natural reserve investigated in the present study is among the lower range, while the overall trematode prevalence in comparable studies ranged from 13.2^56^ to 35.1%^52^. In our system, the snail hosts belonging to the two families Lymnaeidae and Planorbidae harboured the vast majority of trematodes we have identified (92%). This is in line with other broad faunistic surveys (e.g.^57–62^), which indicate that these two snail host families show a particular high prevalence and species richness and can therefore be considered key host groups in aquatic systems^54,63^. Since these trematode-rich key host taxa seem to be prevalent in heavily modified waterbodies, e.g. man-made reservoirs^54^ or fish ponds^52^, we do not find trematode communities with an extraordinary high species richness in the natural reserve compared to other systems, as initially expected.

Overall, with higher free-living diversity in restored habitats and natural reserves, not only does the diversity of suitable trematode hosts increase but also that of non-host organisms which can act as potential diluters of parasites. High free-living diversity can result in various dilution mechanisms, e.g. an encounter reduction of parasites and suitable hosts, a transmission reduction, in which fewer parasites successfully infect a host, or a reduction of reproduction, in which infected hosts produce fewer infectious stages^64^. Experiments under laboratory as well as under natural conditions have shown that dilution effects particularly affect parasites with complex life cycles and with vulnerable larval stages, such as trematodes and other helminths^64–66^. Due to their short-lived and fragile transmission stages (miracidia and cercariae), trematodes are particularly susceptible to reduction by predation or physical disruptions^67–69^. Furthermore, a high density and diversity of non-host species, decoy hosts or alternative hosts, especially benthic organisms, may decrease the probability of transmission stages finding suitable hosts^65^. Miracidia in particular show a high host specificity for their mollusc intermediate hosts^55^, so that high mollusc diversity is likely to reduce trematode transmission to target hosts, while parasite species richness might be less impacted by the diversity of vertebrates, for which trematodes show lower host specificity^64^. The species rich and abundant mollusc fauna detected in our study system (15 species) could thus represent one of the main diluters, causing a lower parasite species richness than initially expected. However, the dilution effect of free-living species diversity on parasitism is not universal and may depend on various factors, such as the individual species composition in a particular habitat, the characteristics of certain host-parasite assemblages, or the scale of the study^65^. To further explore such essential concepts in host-parasite ecology, comparable studies from various aquatic systems and habitats with different species compositions will be required.

However, comparing parasite diversity and functional ecology across different ecosystem often remains hindered and limited by a patchy knowledge of parasite taxonomy, host-specificity, and by dissimilar sampling and study approaches in different regions. For instance, some of the available freshwater studies assessing parasite diversity are broad faunistic surveys, in which many snail hosts, including those with low parasite diversity (e.g*. Potamopyrgus antipodarum*, *Physa acuta*, *Aplexa hypnorum*), were sampled^51^ (present study). Other studies, in contrast, specifically targeted selected species-rich key trematode hosts in the habitat^54,57^. In addition, we find different degrees of resolution regarding parasite identification across studies. Some assessments have focused intensively on selected taxonomic parasite groups using morphological and molecular approaches to discover cryptic species diversity within problematic trematode lineages. Selbach et al.^54^, for example, focused intensively on the genera *Diplostomum* and *Neopetasiger*, whereas Soldánová et al.^57^ examined the species complex in the genus *Plagiorchis* in detail, resulting in the discovery of a high number of species in these groups. Altogether, such differences need to be considered when comparing parasite diversity in different systems, and when using parasites as bioindicators to determine environmental conditions and changes over time and between habitats^35–37^.

Moreover, for many taxa, data on host specificity of trematodes in their second intermediate and final hosts are still too scarce, and many life cycles of parasites are not fully known^70^. The decreasing parasitological taxonomic expertise and the limited number of large-scale studies of vertebrate final hosts are major obstacles in elucidating many parasite life cycles^19^. For instance, parasites considered to be host-specific might be able to infect a wider host range, as host specificity data are usually based on a limited number of samples^71^. On the other hand, taxa considered generalists might be much more host specific. For example, *Diplostomum mergi* was long considered a generalist species until it was recently discovered to constitute a species complex whose individual members are highly host-specific in their final host^72^. Accordingly, the available literature information on final hosts is often designated in rather broad categories, so it may appear that many parasites are not particularly specific with respect to their final host taxa. This is also reflected in the results of our study, which show that most of the parasites detected seem to parasitize very common and widespread host taxa, e.g., waterfowl or fish-eating birds.

Keeping the above-mentioned limitations in mind, the results from our study allow some valuable comparisons with host-parasite communities from other habitats and ecosystems. Even the rather broad final host categories of parasite communities can reveal information on distinctive free-living communities utilized by the parasites in different aquatic ecosystems that represent a characteristic final host composition. For example, despite the close geographic proximity, similar study approaches as well as similar reported trematode species richness and prevalence, the results of Selbach et al.^54^ and from the present study reveal fundamentally different final host structures in these waterbodies (see Fig. 5a vs b). Moreover, the parasites’ life cycle complexity, i.e. the number of hosts required to complete the full life cycle, can serve as an indicator to assess biotic interactions in ecosystems. While parasite species with longer life cycles that rely on trophic transmission can indicate longer food chains and thus more complex biotic interactions in a habitat, a higher share of less complex parasite life cycles can indicate ecosystems that are more severely impacted^73^. Assessments from marine systems and estuarine marshes using parasite communities as bioindicators could show contaminated or only recently restored habitats to contain mostly parasites with short life cycles with only one intermediate host, or direct transmission pathways^74,75^. The natural reserve of the present study is predominantly characterized by trematode species with long life cycles that require two or three intermediate hosts (85% of all species, see Fig. 4). Although the statistical analysis did not reveal any significant differences between the studies compared, the complex life cycles and diverse final host groups of the trematode communities in the present study suggest a diverse and trophically well-connected free-living fauna in the protected nature reserve adjacent to the River Rhine. These long chains of host-parasite connections can indicate stable community structures that provide the basis for ecosystem resilience and persistence^21,76^. Overall, in addition to the diversity of parasites that can indicate ecosystem health^21^, their functional ecology and life histories can provide valuable information on interspecific interactions in these habitats.

In summary, we investigated a protected freshwater ecosystem that provides a valuable model system for free-living and parasite diversity. However, our study also highlights the patchy and heterogeneous data availability, especially with regard to final hosts specificity, which makes it difficult to draw conclusions on the functional diversity and compare parasite communities across various habitats. We therefore encourage future studies on host-parasite communities in natural and protected aquatic systems as well as taxonomic research in this field that will allow a better resolution of cryptic trematode diversity and host specificity for future comparisons. Ultimately, understanding and predicting how global climate change and other anthropogenic pressures will affect complex and fragile freshwater ecosystems and their biotic communities will require a thorough awareness of the parasite communities and their ecological roles that too often remain overlooked in environmental assessments and conservation approaches.

## Materials and methods

In total, 1648 snails of 15 species belonging to seven families were collected and examined for trematode infections during monthly collections in spring (May), summer (June–August) and autumn (September, October) in 2017. Additionally, 346 snails of the species *A. balthica* were sampled monthly from May to September in 2019. To allow comparability, the same sampling effort (time spent sampling) was applied at each sampling site, during each sampling trip, and for all snail host species. Snails were identified to the species level using the identification keys of Glöer^77^ and Welter-Schultes^78^. Snails were collected with hand-nets from the submersed vegetation or picked by hand from sediments, stones, deadwood and macrophytes along the shoreline of the oxbow or the littoral zone of the pond. All snails were taken to the laboratory, placed in individual beakers filled with filtered river water at 20 °C room temperature and exposed to artificial light to induce the emission of cercariae. After the day of sampling, every beaker was screened daily for three consecutive days under a stereomicroscope for the presence of cercariae. Snails that did not show an infection with trematodes during this time were dissected and examined for prepatent infections (rediae/sporocsysts).

### Study site

All snails were collected at two sampling sites at the lower River Rhine (R1: 51° 47ʹ 59.2ʹʹ N 6° 21ʹ 46.3′′ E; R3: 51° 48′ 37.1′′ N 6° 21′ 23.4′′ E) and one groundwater-fed pond of its adjacent floodplain (R2: 51° 49′ 07.0′′ N 6° 20′ 26.8′′ E) in North Rhine-Westphalia, Germany (Fig. 1). Until the year 1800, the Rhine river was largely unaffected. During industrialisation, however, dyking, canalisation and thus the decoupling of backwaters from the main watercourse occurred. The natural reserve Bienener Altrhein is part of the Rhine floodplains between Rees and Emmerich, which are among the few remaining natural floodplains in Europe. They form a unique system of oxbow floodplains, and have been protected since 1969. Various measures have been taken to preserve this area as natural as possible^79^.

### Morphological analyses

Cercariae were identified alive under a light microscope (Olympus BX51) based on identification keys^54,59,60^ and morphological descriptions and other relevant publications (e.g.^70,80–82^). For documentation and further identification photomicrographs of visible features were taken for collected species with an Olympus UC30 digital camera attached to the light microscope.

### Molecular analyses

Cercariae were molecularly spot-checked. DNA was isolated from pooled fixed cercariae by salt precipitation according to Grabner et al.^83^. PCR products were purified (my‐budget PCR/Gel‐purification kits; Biobudget Technologies, Krefeld) and sent for sequencing (GATC Biotech, Constance). Sequences were compared to GenBank entries by Blast‐searches (http://blast.ncbi.nlm.nih.gov/Blast.cgi).

### Data analysis

Trematode prevalence (p) was calculated separately for parasite assemblages in all infected snail species as the proportion of infected host individuals in relation to the total number of host individuals in a population (p = n_inf_/N × 100, where *n*_*inf*_ is the number of infected snails of one species and *N* are all snails of one species in a sampled population) following Bush et al.^84^. The overall prevalence was calculated for all parasites in all snail species as the proportion of all infected host individuals in relation to the total number of all host individuals (Op = a_inf_/S × 100, where *a*_*inf*_ is the number of infected snails of all species and *S* are all snails collected). Species richness was calculated as the total number of trematode species in all host individuals. The relative species richness (S_r_) was calculated for parasite assemblages in all infected snail species as a quotient of the number of trematode species in relation to the total number of infected snail host species studied (S_r_ = t/M, where t is the number of detected trematode species and M are all infected snail species sampled).

### Statistical analyses

Statistical analyses were performed with the open-source software Rstudio (version 2021.09.0, Rstudio Inc.) based on R (version 4.1.1, R Core Team; (www.r-project.org)). A χ^2^ test followed by a post-hoc pairwise test of independence with Bonferroni correction was applied to compare overall trematode prevalence between sampling months. While a Fisher’s exact test was employed to assess differences in the proportions of parasite species with one and two or three intermediate hosts between individual studies.

## Data Availability

The raw data that support the findings of this study are available from the corresponding author, [J.S.], upon reasonable request.
